# Hypercalcemia From Trenbolone Use: A Case Report

**DOI:** 10.7759/cureus.102580

**Published:** 2026-01-29

**Authors:** Kaitlyn C Lizardo, Sarah Merchant, Emily Drone

**Affiliations:** 1 Emergency Medicine, JPS Health Network, Fort Worth, USA

**Keywords:** acute hypercalcemia, acute kidney injury, anabolic-androgenic steroids, clinical case report, trenbolone

## Abstract

In this case, a patient presented to the emergency department (ED) with fatigue after CareNow noted abnormal laboratory results. The patient reported using trenbolone, an anabolic-androgenic steroid (AAS) often used for athletic performance enhancement or cosmetic purposes. In the ED, the patient was noted to have significant hypercalcemia (15 mg/dL) and acute kidney injury (AKI) (creatinine 6.02 mg/dL). The workup ruled out malignancy, hyperparathyroidism, and vitamin D toxicity. The patient was admitted and received intravenous fluids and pamidronate, which improved the patient’s hypercalcemia and AKI. This case adds to the limited literature on metabolic and renal dysfunction associated with AAS use. Additionally, this case highlights the importance of a thorough history, including supplements and nonprescribed substances that patients may be using, as these can contribute significantly to their presentation and care.

## Introduction

Approximately 0.7-2% of patients admitted to the hospital have hypercalcemia noted in their laboratory evaluation [1-3]. In the setting of hypercalcemia, patients most commonly present with neuropsychiatric or gastrointestinal complaints, which can include weakness, lethargy, coma, nausea, vomiting, constipation, or weight loss [1]. The severity of these symptoms often correlates with the acuity of the onset and the level of measured serum calcium [1].

Trenbolone is a synthetic anabolic-androgenic steroid (AAS) often used for its performance-enhancing abilities or cosmetic effects [4]. Approximately three to four million Americans have used AAS in their lifetime, with the highest prevalence in young adult males [5]. The mechanism by which trenbolone and other AAS cause hypercalcemia is not completely known, but there are multiple proposed mechanisms [6,7].

Recognition of the association between AAS use, hypercalcemia, and acute kidney injury (AKI) is critical for timely diagnosis and treatment. This case report aims to highlight the clinical presentation, diagnostic considerations, and pathophysiological mechanisms underlying these complications in the context of AAS misuse, contributing to the growing body of evidence on the renal and metabolic risks associated with performance-enhancing drug use [8-10].

## Case presentation

A 41-year-old man presented to the emergency department (ED) after referral from an urgent care clinic for abnormal laboratory results from the previous day. He was informed that his calcium level and kidney function were abnormal and was advised to seek further evaluation. He reported one week of progressive fatigue and dizziness with positional changes. He described mid-epigastric pain radiating to the sternum associated with nausea without vomiting, as well as darker-than-normal urine. He reported working outdoors but noted difficulty maintaining adequate hydration. The patient denied any medication use at home, including no analgesics. Additionally, he disclosed the use of trenbolone but declined other supplements or steroid use.

**Figure 1:**
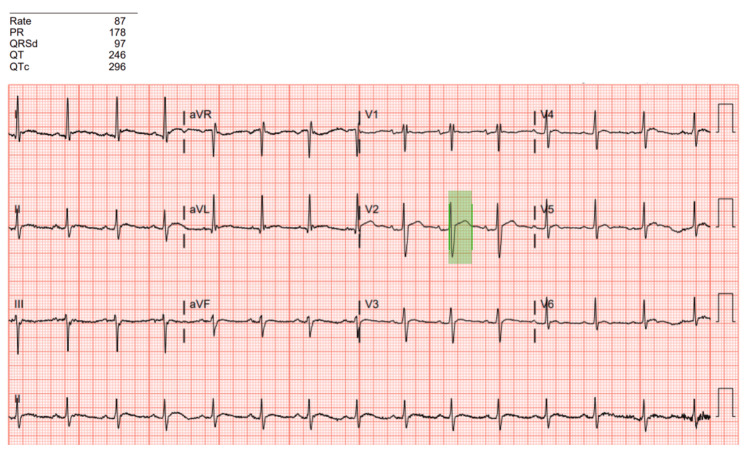
Electrocardiogram demonstrating QT shortening The patient's electrocardiogram obtained on presentation to the emergency department demonstrated an abnormally short QTc (296) likely secondary to hypercalcemia. The QT interval is highlighted in green. EKG, electrocardiogram

Vital signs demonstrated tachycardia with a heart rate of 108; otherwise, vital signs were normal. Physical examination was normal, without focal findings or abdominal tenderness. Laboratory evaluation showed significant hypercalcemia of 15 mg/dL (reference range: 8.7-10.4 mg/dL) with a serum albumin of 2.7 g/dL (reference range: 3.4-5 g/dL). Calcium corrected for albumin was 16.5 mg/dL. Additionally, the patient had elevated bicarbonate >40 mmol/L (reference range: 20-31 mmol/L), blood urea nitrogen of 33 mg/dL (reference range: 9-23 mg/dL), creatinine of 6.02 mg/dL (reference range: 0.70-1.3 mg/dL), creatine kinase of 376 U/L (reference range: 46-171 U/L), aspartate aminotransferase of 92 U/L (reference range: <34 U/L), and alanine aminotransferase of 37 U/L (reference range: 7-49 U/L). Urinalysis showed trace blood with no RBCs, no bilirubin, and no ketones. Parathyroid hormone (PTH) was ordered to evaluate for primary hyperparathyroidism as the cause of hypercalcemia. The PTH level was low at 7.4 pg/mL (reference range: 18.4-90.1 pg/mL), suggesting non-PTH-mediated hypercalcemia. PTH-related peptide was slightly elevated at 3.7 pmol/L (reference range: 0-2.3 pmol/L). An electrocardiogram, noted in Figure 1, was remarkable for sinus rhythm at 86, a QT of 296, and no significant ST changes. A computed tomography scan of the chest, abdomen, and pelvis was performed to evaluate for malignancy and was unremarkable.

**Figure 2:**
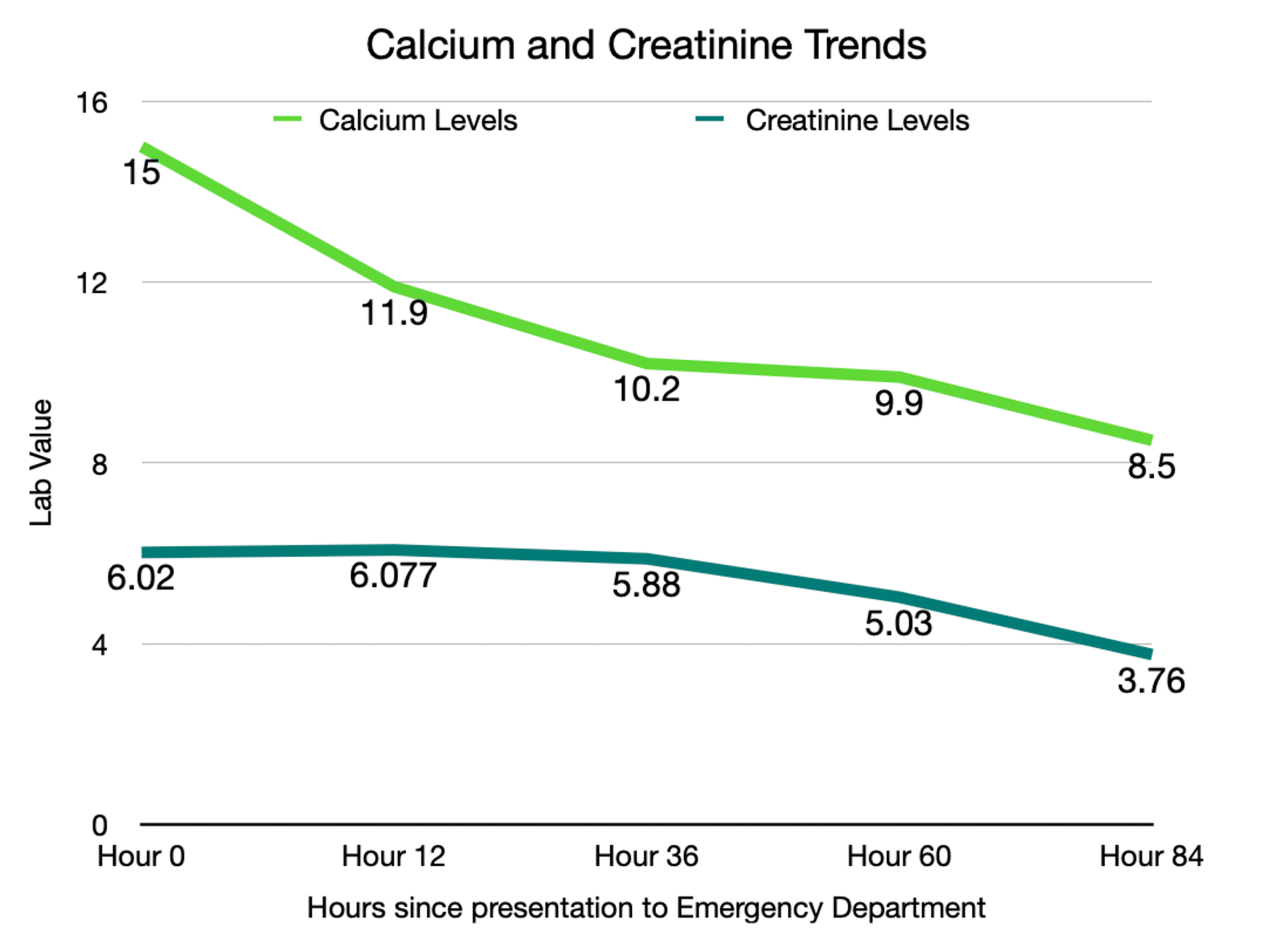
Serum calcium and creatinine trends Serial serum calcium and creatinine levels from hour zero (emergency department presentation) to hospital discharge, demonstrating progressive improvement following initiation of intravenous hydration and pamidronate.

The patient was started on intravenous fluids for treatment of AKI and hypercalcemia and was admitted to the hospital. Nephrology recommended pamidronate, which was started within hours after admission. During hospitalization, the patient had normal 25-hydroxyvitamin D levels with low 1,25-dihydroxyvitamin D, supporting a non-vitamin D-toxicity etiology. Over the next few days, with continued intravenous fluids and pamidronate, the patient’s hypercalcemia and kidney function improved, allowing for discharge from the hospital as noted in Figure 2. After exclusion of malignancy, hyperparathyroidism, and vitamin D toxicity, trenbolone use was the most likely cause of hypercalcemia.

## Discussion

Hypercalcemia is defined as serum calcium above the upper limit of normal, greater than 10.5 mg/dL [2]. This condition can arise from various underlying causes, such as primary hyperparathyroidism and malignancy, which account for approximately 90% of cases. Less commonly, hypercalcemia can be secondary to vitamin D toxicity, drug-related issues, or other endocrine-related disorders [2]. While moderate hypercalcemia (12.0-13.9 mg/dL) may present with constitutional symptoms such as fatigue and constipation, severe hypercalcemia or hypercalcemic crisis (≥14 mg/dL) can often produce acute issues such as altered mental status and cardiovascular membrane instability [2].

Calcium levels in the body are regulated by PTH and vitamin D. About half of the calcium in the body is ionized and biologically active, while the other half binds to albumin and immunoglobulins [11]. PTH is released when ionized calcium levels are low to stimulate calcium release through osteoclast-mediated bone resorption and to increase distal nephron and loop of Henle reabsorption of calcium [11,12]. In the proximal nephron, PTH increases 1α-hydroxylase transcription, which stimulates conversion of 25-hydroxyvitamin D (calcidiol) to 1,25-dihydroxyvitamin D (calcitriol) [12,13]. This results in calcitriol-mediated upregulation of calcium channels and exchangers in the small intestine, increasing absorption [13].

The leading cause of hypercalcemia is malignancy [2,3]. Up to 44% of hypercalcemia is secondary to malignancy, with lung cancer followed by multiple myeloma and renal cell carcinoma [2]. Hyperparathyroidism is the second most common cause of hypercalcemia [1-3].

Less commonly, hypercalcemia may arise from multi-substance use, including vitamin D intoxication, or as a paraneoplastic-like effect of anabolic-androgenic steroids (AAS). AAS use has been implicated in cases of multi-organ dysfunction, including AKI, among bodybuilders [8,9]. The literature describes three cases of hypercalcemia associated with AAS and other supplement use, leading to AKI and systemic complications [8-10]. AAS misuse is increasingly recognized as a cause of diverse renal pathologies, including acute tubular necrosis, focal segmental glomerulosclerosis, and bile nephropathy, often manifesting as AKI [14]. The pathophysiology of AAS-induced renal injury is multifactorial, involving direct nephrotoxicity, hemodynamic changes, and secondary effects from associated practices such as high protein diets, dehydration, and concomitant use of other nephrotoxic agents [14]. Furthermore, AAS-induced hepatotoxicity can lead to cholestatic jaundice and secondary bile nephropathy, further contributing to AKI [14].

Our patient presented with hypercalcemia with no evidence of elevated PTH, malignancy, significant dehydration, or vitamin D toxicity as the primary cause. The suspected source of the patient’s hypercalcemia was secondary to trenbolone use.

As a synthetic AAS, trenbolone has potent anabolic effects and limited androgenic activity [4]. Despite well-recognized performance-enhancing and cosmetic effects, AAS are associated with incompletely characterized multisystem toxicities, with approximately one-third of users experiencing adverse effects, including acne, gynecomastia, hirsutism, hypertension, and cardiac dysrhythmias [4].

The mechanism by which trenbolone causes hypercalcemia is not completely known. Vincieto et al. suggest that AAS may influence calcium levels through two pathways: a non-genomic (rapid) pathway and a genomic (slow) pathway [6]. In the non-genomic pathway, androgens bind to a membrane receptor, activating a signaling cascade that stimulates voltage-dependent calcium channels to release calcium into the cytosol from the endoplasmic and sarcoplasmic reticulum via inositol 1,4,5-trisphosphate receptors [6]. Membrane-bound proteins modulate the efflux of calcium from the cell into the plasma [6]. Androgen receptors in the cytosol bind androgens to activate the genomic pathway, altering the expression or regulation of calcium transporters, pumps, or channels and leading to increased efflux of calcium into the plasma [6].

In addition to these proposed mechanisms, trenbolone may cause hypercalcemia through its anabolic and androgenic effects on bone metabolism. Trenbolone stimulates androgen receptors in bone, leading to increased bone turnover and potential release of calcium into the circulation [7].

Trenbolone may have a direct nephrotoxic effect or may have resulted in AKI secondary to severe hypercalcemia, as calcium is excreted primarily by the kidneys. While dehydration could have been a contributing factor, it was less likely to be the primary cause of significant kidney injury. Laboratory evaluation showed no ketonuria, which is often observed in the setting of dehydration, and the patient’s blood urea nitrogen to creatinine ratio was 5.5, well below the typical threshold of >20 seen with dehydration-induced azotemia.

Our case adds to the limited body of literature describing serious adverse effects associated with AAS use. To date, only three published case reports have described anabolic steroid-induced hypercalcemia. These cases reported multi-organ dysfunction linked to AAS use, specifically testosterone, often in combination with other supplements or substances. Trenbolone, a synthetic analogue of testosterone from the nandrolone group of AAS, possesses approximately three times the affinity for androgen receptors compared with testosterone, producing potent anabolic effects on skeletal muscle and bone [7]. This enhanced activity could also have contributed to the multi-organ failure observed in these patients [8-10].

Our patient reported isolated anabolic steroid use, suggesting that anabolic steroids alone may precipitate clinically significant hypercalcemia. Workup in the ED and upon admission revealed no other obvious cause of hypercalcemia, and AKI improved with supportive care and intravenous fluids during hospitalization. While a small number of cases have been reported in the literature, in addition to our case, this does not establish causality.

A limitation of this case is that the duration, dose, and route of trenbolone use were not available. Additionally, while AAS are implicated in metabolic disturbances, other causes of hypercalcemia, such as vitamin A toxicity, cannot be fully excluded, as a vitamin A level was not obtained. Furthermore, unreported concomitant use of other steroids, supplements, or vitamins may have contributed to the patient’s metabolic and renal abnormalities. These limitations highlight the challenges in evaluating substance-related metabolic derangements.

Our case emphasizes the importance of obtaining a thorough history, including the use of supplements, performance-enhancing agents, and nonprescribed substances, as these may contribute to unexpected metabolic or systemic complications such as hypercalcemia.

## Conclusions

This case highlights a patient with hypercalcemia likely secondary to AAS (trenbolone) use. It underscores the importance of considering a thorough history, including the use of supplements, performance-enhancing agents, and nonprescribed substances, as these can be contributing factors to a patient’s presentation in the ED. As there is limited literature on trenbolone and AAS use, this case report contributes to the literature and increases awareness of complications that may arise from their use.

## References

[1] Ravioli S, Lafranchi A, Exadaktylos AK, Haidinger M, Lindner G (2023). Characteristics and outcome of severe hypercalcemia on admission to the emergency department: a retrospective cohort study. Swiss Med Wkly.

[2] Lindner G, Felber R, Schwarz C (2013). Hypercalcemia in the ED: prevalence, etiology, and outcome. Am J Emerg Med.

[3] Thillainadesan S, Twigg SM, Perera N (2022). Prevalence, causes and associated mortality of hypercalcaemia in modern hospital care. Intern Med J.

[4] Borecki R, Byczkiewicz P, Słowikowska-Hilczer J (2024). Impact of trenbolone on selected organs. Endokrynol Pol.

[5] Sagoe D, Molde H, Andreassen CS, Torsheim T, Pallesen S (2014). The global epidemiology of anabolic-androgenic steroid use: a meta-analysis and meta-regression analysis. Ann Epidemiol.

[6] Vicencio JM, Estrada M, Galvis D (2011). Anabolic androgenic steroids and intracellular calcium signaling: a mini review on mechanisms and physiological implications. Mini Rev Med Chem.

[7] Yarrow JF, McCoy SC, Borst SE (2010). Tissue selectivity and potential clinical applications of trenbolone (17beta-hydroxyestra-4,9,11-trien-3-one): a potent anabolic steroid with reduced androgenic and estrogenic activity. Steroids.

[8] Schäfer CN, Guldager H, Jørgensen HL (2011). Multi-organ dysfunction in bodybuilding possibly caused by prolonged hypercalcemia due to multi-substance abuse: case report and review of literature. Int J Sports Med.

[9] Unai S, Miessau J, Karbowski P, Baram M, Cavarocchi NC, Hirose H (2013). Caution for anabolic androgenic steroid use: a case report of multiple organ dysfunction syndrome. Respir Care.

[10] Khanna P, Khatami A, Swiha M, Rachinsky I, Kassam Z, Berberich AJ (2020). Severe hypercalcemia secondary to paraffin oil injections in a bodybuilder with significant findings on scintigraphy. AACE Clin Case Rep.

[11] Pirklbauer M, Mayer G (2011). The exchangeable calcium pool: physiology and pathophysiology in chronic kidney disease. Nephrol Dial Transplant.

[12] Sato T, Courbebaisse M, Ide N (2017). Parathyroid hormone controls paracellular Ca(2+) transport in the thick ascending limb by regulating the tight-junction protein Claudin14. Proc Natl Acad Sci.

[13] van de Graaf SF, Boullart I, Hoenderop JG, Bindels RJ (2004). Regulation of the epithelial Ca2+ channels TRPV5 and TRPV6 by 1alpha,25-dihydroxy Vitamin D3 and dietary Ca2+. J Steroid Biochem Mol Biol.

[14] Davani-Davari D, Karimzadeh I, Khalili H (2019). The potential effects of anabolic-androgenic steroids and growth hormone as commonly used sport supplements on the kidney: a systematic review. BMC Nephrol.

